# The Development of Optomechanical Sensors—Integrating Diffractive Optical Structures for Enhanced Sensitivity

**DOI:** 10.3390/s23125711

**Published:** 2023-06-19

**Authors:** Faolan Radford McGovern, Aleksandra Hernik, Catherine Grogan, George Amarandei, Izabela Naydenova

**Affiliations:** 1 School of Physics, Clinical & Optometric Sciences, Technological University Dublin, D07 ADY7 Dublin, Ireland; c16342516@mytudublin.ie (F.R.M.); aleksandra.hernik@tudublin.ie (A.H.); catherine.grogan@tudublin.ie (C.G.); 2 Centre for Industrial & Engineering Optics, Technological University Dublin, D07 ADY7 Dublin, Ireland; 3 The Group of Applied Physics, Technological University Dublin, D07 ADY7 Dublin, Ireland

**Keywords:** optomechanical, cantilever, MEMS, fiber Bragg, diffractive element

## Abstract

The term optomechanical sensors describes devices based on coupling the optical and mechanical sensing principles. The presence of a target analyte leads to a mechanical change, which, in turn, determines an alteration in the light propagation. Having higher sensitivity in comparison with the individual technologies upon which they are based, the optomechanical devices are used in biosensing, humidity, temperature, and gases detection. This perspective focuses on a particular class, namely on devices based on diffractive optical structures (DOS). Many configurations have been developed, including cantilever- and MEMS-type devices, fiber Bragg grating sensors, and cavity optomechanical sensing devices. These state-of-the-art sensors operate on the principle of a mechanical transducer coupled with a diffractive element resulting in a variation in the intensity or wavelength of the diffracted light in the presence of the target analyte. Therefore, as DOS can further enhance the sensitivity and selectivity, we present the individual mechanical and optical transducing methods and demonstrate how the DOS introduction can lead to an enhanced sensitivity and selectivity. Their (low-) cost manufacturing and their integration in new sensing platforms with great adaptability across many sensing areas are discussed, being foreseen that their implementation on wider application areas will further increase.

## 1. Introduction

Sensors play a fundamental role in almost every aspect of modern life. Across numerous fields from surveillance and security to medical health-care settings, sensors form an integral role in the operation of complex systems and devices being fundamental to the quality and improvement of services and industries. The desire to improve sensing devices focuses on addressing the quality of key parameters—sensitivity, selectivity, response time, stability, drift, range, reproducibility, and cost. Sensors can be described, broadly, as fitting into one of three categories of transducer—mechanical, optical, or electrical [1,2,3]. In recent decades, the combination of these transducing principles has garnered significant interest. By coupling two or more sensing principles, it is possible to enhance the sensitivity when compared with the resolution offered by the individual technologies upon which the sensor configurations are based. Perhaps the most popular category of such devices is microelectromechanical systems (MEMS). Since the latter half of the 20th century, MEMS devices have seen significant commercial applications [4] as well as attracting significant research interest [5,6,7]. MEMS sensors are based on combining electrical and mechanical transducing fundamentals resulting in micromachined devices that are generally on the micro/millimeter scale [8]. Many configurations of MEMS sensors have been presented, including electrochemical, acoustic, and photonic [9]. While such devices have been a catalyst for advancing sensing techniques over the past 20 years, new sensor configurations are continuously sought to address challenges such as fabrication cost and complexity or to meet the demanding requirements of an increasingly complex technological environment. The combination of optical and mechanical transducers to form optomechanical sensors is one such configuration. Much like the MEMS sensors, research into optomechanical sensors has resulted in many configurations and applications [10,11,12].

Traditional optomechanical sensors are typically based on a classical mechanical sensing platform (e.g., cantilever sensor). The mechanical response of this platform is coupled with an optical component that enhances the device’s sensitivity by modulating the properties of a light wave entering the sensor [13]. The key advantage of optomechanical sensors is enhancing the resolution of classical mechanical sensors by the addition of an optical output; the sensitivity and dynamic range of the optical output is also enhanced by the coupling. These devices are of particular interest due to their insensitivity to electromagnetic fields, high resolution, low cost, adaptability, and low power consumption [11]. In general, optomechanical sensors can be divided into groups, such as the following: Fiber optic sensors, Fiber Bragg Grating (FBG) sensors, photonic crystal sensors, mass spring and cantilever configurations sensors, and quantum devices such as cavity optomechanical sensors [14,15,16,17,18,19]. Further categories of optomechanical sensors include nano optomechanical sensors (NOMS) [20] and micro-opto-electromechanical (MOEMS) sensors [21]. While significant research interest into optomechanical sensors has been demonstrated, these devices have yet to achieve significant commercial development.

Diffraction gratings have been demonstrated as high-resolution sensors [22,23], including holographic optical elements (HOE) [24,25], as they offer several advantages, e.g., a simple operating principle with relatively well-understood theoretical responses, simple/well-established fabrication processes, and compatibility with classical mechanical transducing techniques. This lends such devices to offer adaptable, highly commercializable sensors with low-cost production for sensing across disciplines. In this perspective, we will establish the potential for optomechanical sensors in general to offer high sensitivity solutions to modern sensing challenges. The focus will be on coupling of the classical mechanical sensors with diffractive optical structures as these devices offer significant potential to form the next generation of state-of-the-art sensors.

**Figure 1:**
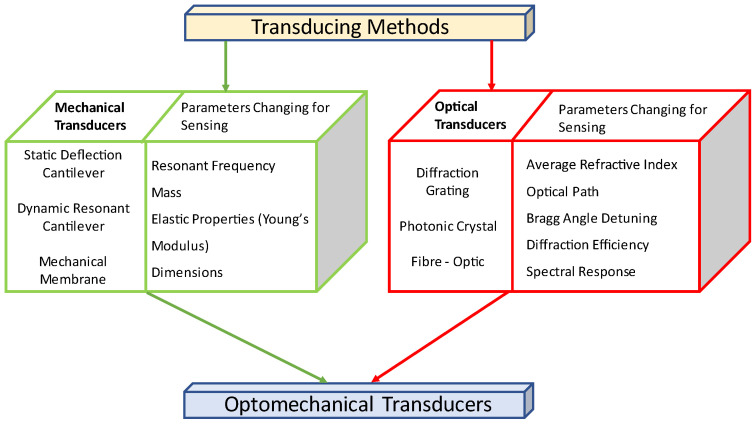
Foundations of optomechanical sensors. The typical response and parameters used by such sensors are presented, various combination being implemented.

Research into optomechanical sensors based on the combination of a classical optical and mechanical transducing method dates to at least the 1970s and 1980s with continuing interest through the 1990s and early 2000s through the advent of the prevalence of MEMS-type devices [26,27,28,29]. Figure 1 presents the broad philosophy behind such sensors, based on the combination of technologies.

Classical optomechanical sensors are typically based on the addition of a classical optical sensing technique to a mechanical base, while other sensors (e.g., FBG) are more fundamentally optical with mechanical actuation incumbent in response to the target analyte [30]. Nano-optomechanical (NOMS)- and micro-optoelectromechanical (MOEMS)-type optomechanical sensors are primarily electrical transducing devices coupled with an optical method of sensing, requiring more complex readout equipment. Quantum sensors, including cavity optomechanical devices that operate based on Stokes and anti-Stokes scattering [31] likewise, can be also considered as a different category.

Table 1 presents a range of applications of classical optomechanical sensors. In each example, the sensor is based on a combination including either a classical optical (e.g., diffraction grating) or classical mechanical (e.g., cantilever) transducer. Sensors based on quantum effects are not included in this table.

Table 1 demonstrates the utility of cantilever-based sensors, combined with an optical sensing technique, in detecting multiple analytes. Cantilever sensors are well researched, with many established fabrication and functionalization techniques [38,39]. Some optical sensing devices have also demonstrated enhanced sensitivity when the target analyte induces a mechanical change. The type of sensors discussed in this perspective are simple in principle. A diffractive optical sensor is combined with a classical mechanical sensor. Electromagnetic insensitivity is maintained while device resolution can be greatly enhanced. These classical diffractive optomechanical sensors have attracted significant interest because of their simpler sensing principles and highly adaptable properties. In the next sections, we discuss the foundations of these sensors.

## 2. Mechanical Sensors

Mechanical sensors describe an innumerable list of sensor types with a large panoply of applications, e.g., accelerometers, flow meters, temperature sensors, strain sensors, and pressure sensors [40]. Mechanical sensors can be actuated magnetically, electrically, thermally, and electrochemically [40,41,42,43]. In broad terms, a mechanical sensor is one that detects a change in deformation, be it a compressive, shear, torsional, contact bending, or frictional force. In this section, we discuss the types of mechanical transducer that are commonly paired with diffractive optical sensors to form optomechanical devices.

### 2.1. Cantilever Sensors

**Figure 2:**
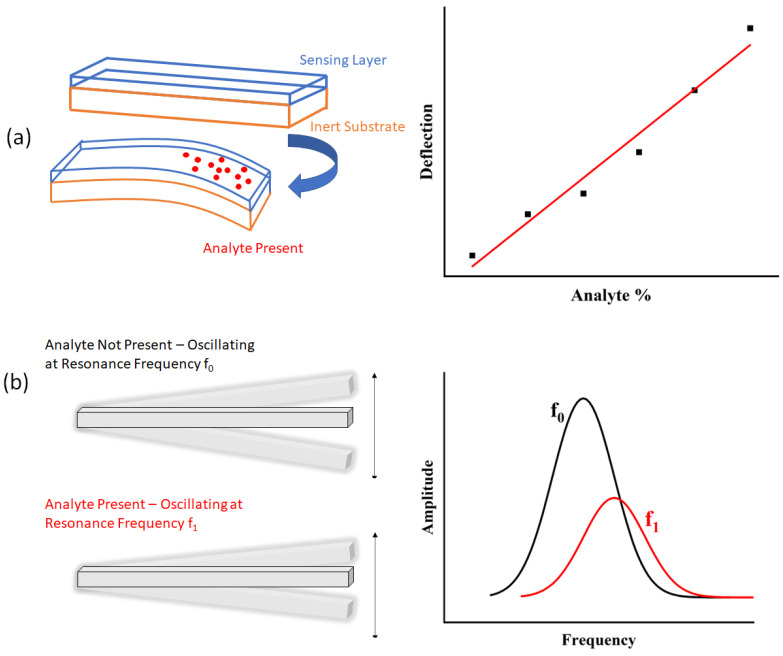
Cantilevers can operate in (**a**) static or (**b**) dynamic mode. In static mode, the cantilever bends due to a differential stress induced between the layers in the presence of the analyte. This deflection is measured as the means of sensing. In dynamic mode, the cantilever, magnetically or electrically actuated, oscillates at its natural frequency, but in the presence of the analyte the resonance frequency changes.

The primary mechanical transducer used in the formation of classical optomechanical sensors is the cantilever. In the classical configurations, the cantilever sensors operate in dynamic and/or static mode, as described in Figure 2.

Static deflection-mode cantilevers are based on the deflection of the cantilever beam due to the presence of the target analyte [44]. In the case of macro size (i.e., from few mm to few cm) cantilevers, this deflection can be measured visually with minimal (if any) visual aids. For microcantilevers, this deflection is measured optically using the ‘optical lever’ method (described later in this section), but other methods (e.g., capacitive measurements) can be employed.

Static deflection-mode cantilevers are typically using a bimaterial configuration, with the deflection of the cantilever a result of the stress differential between the sensing layer and bulk substrate layer as described by the Stoney equation [44].

(1) $$\frac{1}{R}=6\left(\frac{1-\nu }{Et^{2}}\right)(∆s_{1}-∆s_{2})$$

where ν is the Poisson’s ratio, *E* is the elastic modulus of the substrate, *t* is the thickness, *R* is the resultant radius of curvature, and $\left(∆s_{1}-∆s_{2}\right)$ is the difference in the change in surface stress between the two layers. The resultant deflection of the cantilever is then measured as the means of sensing.

Dynamic-mode cantilevers are based on a change in the resonance frequency of a vibrating cantilever due to an increase in mass when the target analyte is present. The mass of the analyte detected can be related to the change in resonance frequency by Equation (2) [44].

(2) $$m=\frac{k}{4}\left(\frac{1}{f_{1}^{2}}-\frac{1}{f_{0}^{2}}\right)$$

where *m*, is the added mass, *k* is the spring constant, and $f_{1}$ and $f_{0}$ are the cantilever resonance frequencies before and after the analyte is present. The typical optical detection methods are the same as for the static method, but the electronics behind the readout becomes more complex.

Table 2 summarizes a few examples of cantilever sensors operating in both static and dynamic mode. The trace explosives sensor presented by Chen et al. [45] and the adipocyte temperature sensor developed by Sato et al. [46] are both based on bimaterial cantilevers. In each case, the target analyte results in a static deflection of the cantilever which is used as the means of sensing. The Chen et al. sensor is based on a siloxane sensing bilayer while the Sato et al. device is fabricated with silicon nitride and gold layers. The detection of the Chen et al. sensor is based on the piezoresistive output of the cantilever. Differential material response cantilever sensors are based on the static mode of cantilever sensing, i.e., the cantilever bending in response to presence of the target analyte [47].

Cantilever sensors are commonly presented in an array format as this can increase the sensitivity and selectivity of such sensors. For example, the gas sensor presented by Wu et al. [48] is based on static deflection-mode cantilevers—in this case, an array of cantilevers being used with the end of each cantilever being functionalized for a specific gas analyte. Cantilever array sensing devices have been demonstrated for numerous sensing methods including optical readout and dynamic and static deflection [51]. Such devices have the advantage of specificity in environments with the presence of multiple analytes as each element of the cantilever array can be functionalized to specific target analytes as desired [51]. The devices presented by Vančura et al. [49] and Wasisto et al. [50] are dynamic-mode cantilever sensors. Such sensors are based on the change in the cantilever resonance frequency in the presence of the target analyte [51].

Table 2 demonstrates the popularity of cantilever sensors as sensing platforms. The underlying principles behind static and dynamic-mode cantilevers is well understood and functionalization of these platforms has been demonstrated for numerous analytes [52,53]. The first way in which this enhancement can be achieved is through measuring the deflection of the cantilever using laser light. Should the cantilever have a reflective surface, the position of the reflected beam can be measured as the cantilever deflects using a position dependent photodetector [44]. This technique, called an optical lever, is used in AFM imaging. In a basic sense, this is a primary version of cantilever optomechanical sensing. However, in this configuration, it is really only the measurement of the cantilever position that is being enhanced, rather than the fundamental device sensitivity. In ‘true’ optomechanical sensors, the optical mode is an independent sensing method which is itself enhanced by its coupling with a mechanical transducer. Cantilever sensors operating in both static and dynamic mode have been coupled with a number of different diffractive optical sensors resulting in enhanced sensitivity. Such devices are discussed in Section 4.

### 2.2. Membrane Sensors

**Figure 3:**
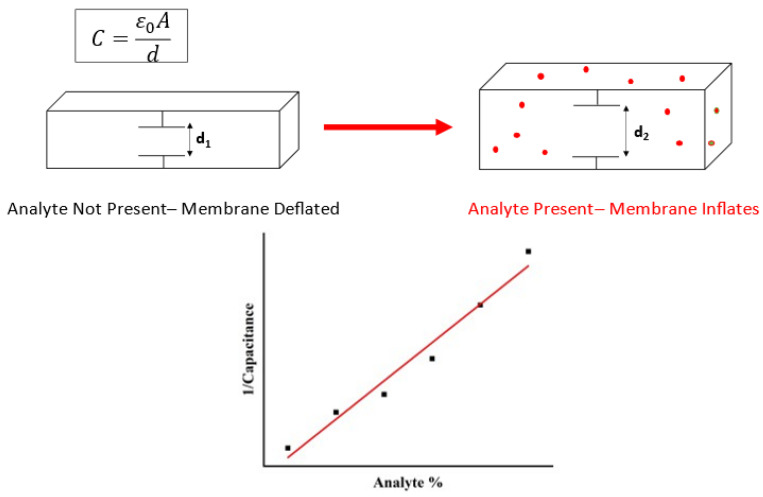
Operating principle of capacitive mechanical membrane sensors. As the membrane inflates with the presence of the analyte, the change in capacitance in measured as the means of sensing.

Membrane sensors have been used as detectors of many analytes with particular interest in pharmaceutical applications [54,55]. Often, membranes operate as electromechanical or electrochemical sensors [56]. There are several sensing mechanisms used in membrane sensing. The membrane can be used as a selective protective layer that allows only the analyte of interest to pass through to the sensing element [57]. A more direct mechanical sensing method is coupling the physical inflation of the membrane with an electrical response. In such devices, the membrane acts as a capacitive type of sensor, where the membrane response to the analyte leads to a change in the electrical output [58,59,60]. An example of the operating principle of a capacitive membrane sensor is illustrated in Figure 3.

Such devices have been demonstrated as effective sensors for numerous analytes as detailed in Table 3.

As demonstrated in Table 3, the combination of a mechanical membrane response with an electrical output is used for detecting a number of analytes. Separate to this method, however, is the use of a membrane as a barrier as demonstrated by Drobek et al. [61]. One of the primary challenges of gas sensing is selectivity; by encapsulating a ZnO nanowire sensor based on resistive changes in a ZIF-8 molecular sieve membrane, Drobek et al. achieve selectivity for H_2_ and a negligible response to C_7_H_8_ and C_6_H_6_ [61]. While this configuration of membrane sensor is of significant interest in gas sensing, it is not a mechanical membrane sensor in a pure sense. The membrane here is acting more as a filter for a sensor, rather than directly as the sensitive element.

The electromechanical type of membrane sensors are examples of more direct membrane sensing techniques. One such method, as described by Tran et al. [62], is based on a membrane inducing a piezoresistive response. When a pressure is applied to the membrane, a stress results in piezoresistors. Several different configurations were examined. In terms of the mechanical response, Tran et al. have demonstrated that by changing the structure of the diaphragm, a difference in the sensitivity, nonlinear error, and deflection are observed [62]. Capacitive-type membrane sensors have also been evaluated for both pressure [63] and humidity [64]. Yang et al. [63] have developed a capacitive sensor for pressure based on a polyvinylidene fluoride (PVDF) nanofiber membrane. When pressure is applied to the system, the distance between the electrodes decreases, leading to a change in the output capacitance [63]. The humidity sensor presented by Hernández-Rivera et al. [64] is based on a similar principle. Here, instead of the distance between the electrodes changing, the presence of moisture leads to a change in the dielectric constant of the membrane [64]. Özbek et al. have presented a membrane sensor based on a potentiometric analysis [65]. This method is based on a chemical reaction between the membrane structure and the drug analyte of interest [65] resulting in a change in the potentiometric output. The sensor exhibited good selectivity, with a linear response for the target Levetiracetam drug and a 25 s response time [65].

A number of different sensing techniques using membrane structures have been presented. Some such as the gas sensor described by Drobek et al. are based on membranes operating as filters for selectivity. Of more interest to this perspective are the mechanical capacitive and resistive type of membrane sensors [62,63,64]. Such sensors are essentially electromechanical devices with a mechanical deformation, or material change resulting in an alteration in the capacitance or resistance of the device. Membranes have also been coupled with optical sensing methods [66,67,68] to form optomechanical sensors.

In this section, three mechanical sensing principles have been discussed—static deflection cantilevers, dynamic resonance cantilevers, and mechanical membrane sensors. While all three platforms have demonstrated highly sensitive characteristics and functionalization for multiple analytes, they are also compatible for coupling with optical sensing methods. This coupling results in enhanced sensitivity. In the next section, optical sensors, often coupled with these mechanical platforms to create optomechanical sensors, are discussed.

## 3. Diffractive and Interferometric Optical Sensors

Like the term mechanical sensor, optical sensors could be used to describe many categories of device with applications for many analytes. With respect to feasibility for coupling with classical mechanical sensors, holographic diffraction, fiber optic, and photonic crystal sensors are of particular interest.

### 3.1. Diffractive Holographic Sensors

**Figure 4:**
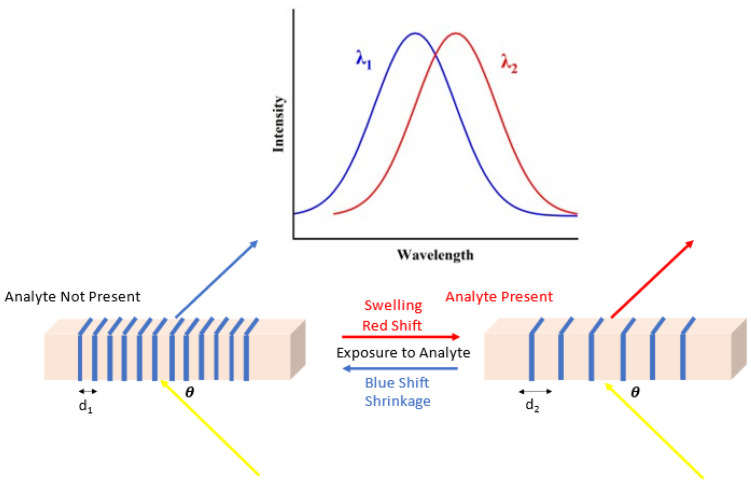
Operating principle of a transmission holographic sensor (where *d* is the grating spacing and θ is the angle of the incident beam on the grating). In this particular sensor configuration, when the analyte is present, the grating spacing changes leading to a change in the peak diffracted wavelength and the intensity of the diffracted beam.

Prime among current state-of-the-art diffractive optical sensors are holographic sensors. Holographic diffraction gratings are formed by the interference of two coherent laser light beams on a photosensitive material. The resultant recording of the interference pattern acts as a diffraction grating with tunable characteristics based on material and recording conditions. Holographic sensors have been demonstrated for numerous analytes such as temperature [69], humidity [70], pH [71], water [72], and gasses [73]. Holographic sensors are based on the change in the properties of the hologram when exposed to the analyte and the analysis of the resultant variation in the hologram’s diffractive properties [25]. The variation in diffractive properties can be achieved either through a dimensional change or change in the average refractive index of the material in which the hologram is recorded or a change in the refractive index modulation of the material, i.e., sensing through the creation of a diffractive structure [25]. These changes in the hologram and resultant variations in its diffractive properties have been applied in a number of different ways. For example, sensors have been developed based on the swelling/de-swelling of the hologram in response to the target analyte [73]. Sometimes this response is intrinsic to already well-established recording media [74] while in other cases the inclusion of additional components is required to achieve the desired change in material volume, such as the doping of photopolymer with nanoparticles [75]. Figure 4 demonstrates the principle of operation of a transmission mode holographic sensor.

Regardless of the method, the introduced change in the volume of the hologram allows for the detection of the target analyte through the resultant variation in the diffractive properties of the grating, i.e., diffraction efficiency and/or peak wavelength shift. The method of sensor readout generally requires the probing of the holographic grating with a beam of light and power meter as a minimum; however, a more simplistic sensing method based on color change of the grating has been presented [74]. Table 4 lists examples of holographic diffraction sensors.

Table 4 demonstrates the wide range of analytes detectable using holographic sensors. Several clear trends develop in that the operating method of each sensor is a volume change in the holographic material, resulting in a change in the spacing of the holographic diffraction grating and in some examples a change in the refractive index modulation [76]. In some cases, this swelling is an intrinsic property of the holographic material in the presence of the analyte in question [77,80], while in other examples, a dopant must be included in the holographic material to elicit the desired sensing response [76]. It has also been demonstrated that the doping of a non-photopolymerizable material allows for the formation of an analyte sensitive holographic grating [78]. The use of a dopant while increasing the potential utility of a holographic recording material can require a more complicated and possibly more costly fabrication process. The resultant change in grating spacing in response to the analyte of interest produces a shift in the peak diffracted wavelength; this effect is demonstrated in both reflection and transmission holographic gratings. This change in peak wavelength can be quantitatively measured and calibrated as a means of sensing in addition to a visual readout (hologram color change). While holographic sensors offer accurate low-cost sensing solutions in many cases, challenges are still present in the optimization of such devices. Some holographic materials, acrylamide photopolymer for example, suffer from disadvantages of cross-sensitivity [76,79], making the fabrication of a sensor with high specificity challenging. Furthermore, zeolite nanoparticle-doped materials can enhance the specificity of a holographic sensor, but such systems can have problems with respect to sensor reversibility [82]. Finally, holographic sensors often require high-stability environments for accurate and repeatable operation, making the development of commercial-grade environmental holographic sensors challenging.

Despite this, holographic sensors offer significant potential due to their diverse sensing ability, well-understood diffraction properties, low-cost fabrication techniques, and potential for functionalization. The incorporation of holographic sensing principles and materials in optomechanical sensing devices has seen growing interest in recent years due to the potential to provide enhanced sensitivity over traditional holographic sensors, while addressing issues of specificity and stability. The simple diffractive structures that form holographic sensors significantly enhance the resolution of mechanical transducers. In turn, the mechanical response of the transducer is generally more significant than the physical changes experienced by holographic sensors alone. These exaggerated physical changes result in enhanced sensitivity of the optical mode of sensing as well. In this sense, the coupling of holographic diffractive optical-type sensors with classical mechanical sensors is a symbiotic combination, greatly enhancing the sensitivity of the individual technologies alone.

### 3.2. Optical Fiber Sensors

**Figure 5:**
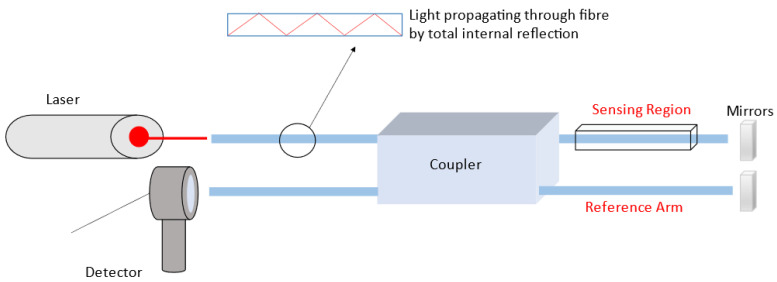
Operating principle of a transmission an interferometric fiber optic sensor. Light propagates through the optical fiber by total internal reflection. One arm of the coupler acts as a reference while the other is the sensing arm. The propagation of light in the reference arm is modulated by the presence of the analyte which changes the interference signal received by the detector.

Optical fiber sensors are mainly based on one of the following two sensing techniques—the wavelength (spectroscopic) interrogation or the intensity interrogation [83]. Regardless of the technique, the core principle of fiber optic sensing is to detect a difference in the input and output light (in terms, usually, of intensity or wavelength) from the fiber depending on the presence of the target analyte [84]. Fiber optic sensors operate on the principle of total internal reflection (TIR) at the fiber wall, the concept in terms of a sensing device being that in the presence of an analyte the wall properties (volume, refractive index, conductivity) change, resulting in a variation in the optical output [84]. A common configuration of optical fiber devices is that of an interferometric sensor. The fiber is split into two beams, one of which acts as a reference beam, while the other beam is directed through the analyte of interest. This results in a variation between the beams’ optical paths, causing a constructive or destructive interference, the extent of which is measured as the means of sensing [85]. This method, called in-fiber interferometric sensing, uses a number of different techniques including Fabry–Perot, Mach–Zehnder, Michelson, and Sagnac interferometry [84]. Figure 5 demonstrates the operating principle of fiber optic-type sensors.

Fiber optic sensing devices have been demonstrated for multiple analytes as illustrated in Table 5.

**Figure 6:**
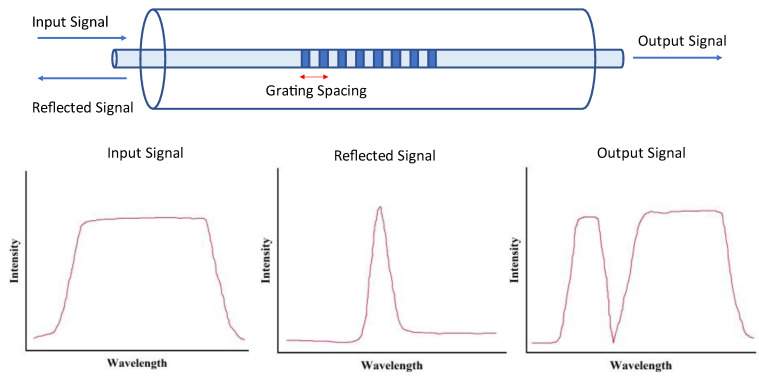
FBG operating principle. When the FBG is in contact with the analyte of interest, their grating spacing changes, resulting in a shift in the peak wavelength of the reflected signal.

The basic principle of interferometric transducers can be applied to numerous analytes demonstrating an adaptable sensing platform. Furthermore, optical fiber sensors are flexible, lightweight, and robust [92]. They also have the possibility to act as compact/miniaturized sensors with fibers of diameter less than 300 μm being already demonstrated [93]. Furthermore, their electromagnetic insensitivity gives them advantages over MEMS and MOEMS type devices, particularly in health-care settings where devices generating magnetic and electric fields are abundant [94]. Much like holographic diffractive sensors, optical fiber devices have significant potential for enhanced sensitivity through coupling into optomechanical sensors. Fiber Bragg Grating (FBG) sensors have been demonstrated as effective optomechanical sensors. Figure 6 demonstrates the operating principle of an FBG sensor.

FBG are Bragg gratings inside optical fibers [95]; they reflect light of a certain wavelength depending on the refractive index modulation of the grating, or the grating period, both of which can be changed by the presence of a target analyte [96]. FBG sensors have been demonstrated as effective optomechanical sensors, including coupling with cantilever transducers for enhanced sensitivity [97,98]. The flexible nature of fiber optic sensors lends them well to coupling in an optomechanical configuration. Similar to holographic diffractive sensors, this coupling leads to an increase in sensitivity and increases the adaptability of the sensing platform.

### 3.3. Photonic Crystal Sensors

**Figure 7:**
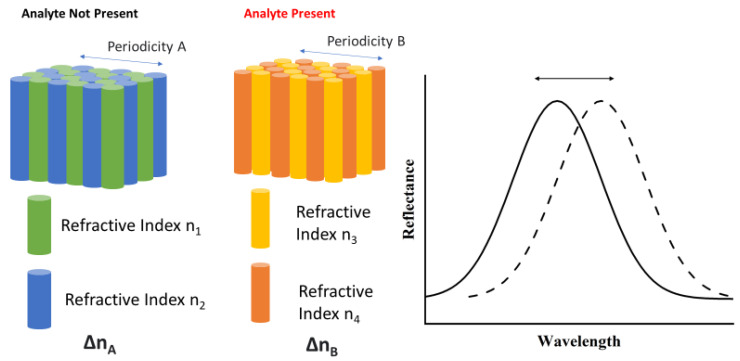
Operating principle of a 2D Photonic Crystal Sensor. The presence of the analyte causes a change in the refractive index modulation and/or the periodicity of the crystal. This results in a shift in the peak wavelength of reflected light.

Photonic crystals are materials with a high degree of order—i.e., a regular arrangement of their constituent matter, where the dielectric constant of the material is modulated periodically [99]. The periodic arrangement of photonic crystals is on the scale of visible light and, therefore, they influence its propagation [100]. Variations in the refractive index or period of the photonic crystal will alter the properties of the propagating light and can be utilized as a sensing mechanism. A number of different photonic crystal sensing techniques have been developed, with applications in chemical, humidity, biological, gas, oil, and temperature sensing demonstrated [101]. Several different sensing techniques have been demonstrated using photonic crystals, including refractive index-based sensors and optical absorption-based sensors [102]. A schematic detailing the operating principle of photonic crystal sensors is seen in Figure 7.

Photonic crystal sensors can be fabricated in a 1D, 2D, and 3D configuration, with 2D being the most commonly used [103]. Table 6 summarizes examples of photonic crystal sensors for various analytes.

The methane sensor presented by Ashraf et al. [104] is a 2D photonic crystal with a cavity made from cryptophane E made of two slots and holes in the middle row of the crystal structure [104]. Methane, the analyte of interest, alters the refractive index of the cryptophane E, resulting in a change in the resonant wavelength of the system [104]. The hydrogen sulfate sensor presented by Afsari et al. is based on a similar working principle [107]. Here, the slot cavity is coated with tungsten oxide. In this case, when hydrogen sulfide is present, the refractive index of the tungsten oxide coating changes, resulting in a shift in transmitted wavelength of the sensor [107]. Such photonic crystal sensors based on variations in the refractive index and, as a result, a shift in the output transmitted wavelength of the sensor, have been demonstrated as effective sensors for numerous analytes. The pH sensor presented by Fenzl et al. [105] works slightly differently. In this case the refractive index is changing in the presence of the analyte (the authors also note the change in lattice constant). However, here, the reflected light is measured using reflection spectroscopy [105]. Depending on the ionic strength of the analyte, the photonic crystal displays a different color [105]. The sensor developed by Fenzl et al. [106] is also based on measuring variations in the wavelength of reflected light. In this sensor, polydimethylsiloxane (PDMS), an inert polymer, has 173 nm monodisperse polystyrene nanoparticles incorporated into its structure [106], thus forming a photonic crystal. Depending on the solvent the sensor is placed in, there is a change in color observed. The reflected wavelength was observed to vary in a linear manner, depending on the concentration of analyte present [106]. Photonic crystal sensors have also been utilized in the detection of biological samples as demonstrated by Ayyanar et al. [108]. Their work presents a photonic crystal sensor for detection of cancer cells—cervical, breast, and basal [108]. Their device is based on the selective infiltration method, with the samples in fluid form infiltrated into the cavity resulting in a variation in the loss and transmission wavelength spectrum due to a change in refractive index [108].

Photonic crystal sensors are diffractive optical sensors with demonstrated utility as an effective and adaptable sensing platform, based on a change in the refractive and lattice properties of the crystal in the presence of the analyte of interest. While operating as optical sensors in isolation achieves high sensitivity, examples have been put forward of photonic crystal sensors coupled with mechanical transducers for enhanced sensitivity [109].

## 4. Diffractive Optomechanical Sensors—Enhanced Sensitivity through a Combination of Technologies

Thus far, the applications of several different mechanical and optical sensing techniques have been discussed. Cantilever sensors, membrane sensors, holographic/diffraction grating sensors, fiber optic sensors, and photonic crystal sensors have been demonstrated not only as highly effective and adaptable devices but also as having significant potential for enhanced sensitivity through coupling into an optomechanical configuration. Table 7 lists examples of such classical diffractive optomechanical sensors, demonstrating their prevalence as adaptable sensing platforms across numerous fields.

**Figure 8:**
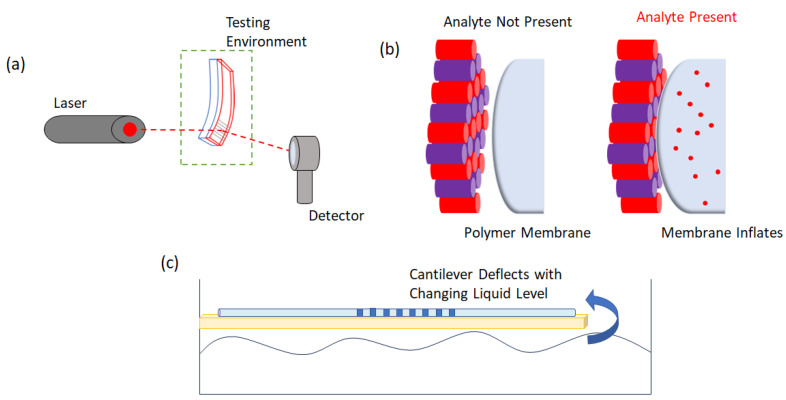
Operating principle of several diffractive optomechanical sensors. (**a**) Static deflection cantilever sensor combined with a diffraction grating. This is similar to the sensor presented by Grogan et al. [110] where diffraction efficiency of the grating changes depending on the angle of deflection of the cantilever with varying amount of analyte present. (**b**) Photonic crystal sensor combined with an inflating membrane similar to the pressure sensor presented by Nazirizadeh et al. [121] where the inflating of a membrane results in contact with a photonic crystal changing the optical output. (**c**) An FBG combined with a static deflection cantilever similar in concept to that presented by Guo et al. [118] (different position of FBG on cantilever) where the changing liquid level results in a deflection of the cantilever and a variation in the output of the FBG.

As demonstrated, a number of different combinations of diffractive optical and mechanical transducers have been implemented. The operating principle of selected configurations discussed in Table 7 are depicted in Figure 8.

Among the multiple combinations of sensor that have been utilized, the cantilever-based diffractive sensors have garnered most interest, likely due to their highly controllable and adaptable sensing properties. Thus, cantilever mechanical transducers have been coupled with diffraction gratings as well as fiber-optic/FBG sensors. An example of a diffraction grating-style cantilever sensor is the humidity sensor presented initially for the first time by Grogan et al. [110]. In this case, a holographic diffraction grating is coupled with a bimaterial static deflection cantilever [110], i.e., an inert PDMS layer coated with a hydrophilic photopolymer. With increasing the relative humidity, the differential strain between the layers results in a deflection of the cantilever [110]. This deflection can be measured by eye to a detection limit of approximately 1% relative humidity. Coupling with a volume holographic diffraction grating, recorded in the photopolymer layer allows for an approximately ten-fold increase in sensitivity over the mechanical method alone [110]. The detection is based on measuring the varying intensity of the diffracted beam from the grating, as the angle of cantilever deflection changes with increasing/decreasing relative humidity [110]. A similar configuration of device was later presented by Yu et al. [111]. In this case, the acrylamide photopolymer was coated on a flexible substrate, with a volume transmission grating recorded in the photopolymer layer [111]. The Yu et al. cantilever was tested for both bilateral and unilateral bending with the peak diffracted wavelength shift measured as means of detection [111]. While the device has not been functionalized, the bending of the cantilever beam can be detected with high sensitivity with a linear dependence of the peak wavelength on deformation observed. Furthermore, the sensor can provide information on the distribution of the bending stress applied [111]. While the Grogan et al. and Yu et al. sensors are based on photosensitive recording materials with holographically formed diffraction gratings, diffractive cantilever sensors were developed using more traditional cantilever materials. Thus, the devices presented by Yaralioglu et al. and Sarioglu et al. are silicon AFM cantilevers with micromachined diffractive fingers for enhanced sensitivity [112,113]. The cantilever presented by Yaralioglue et al. is an AFM contact-mode cantilever sensor [112]. The silicon cantilever consists of two micromachined diffractive fingers, one including the tip, which deforms during scanning, and one connected to the cantilever support, which remains rigid [112]. The illumination of these gratings results in a diffraction pattern and the intensity of the various diffracted orders depends on the deflection of the cantilever [112]. The authors conclude that this diffractive optical coupling with a classical AFM cantilever configuration leads to enhanced sensitivity when compared to a traditional optical lever sensing method as the diffractive finger device is insensitive to vibration and its sensitivity does not depend on cantilever length [112]. A similarly micromachined diffractive grating fingers method is employed by Sarioglu et al., except this time for use with tapping mode AFM [113]. In this case, the cantilever is excited at 22 kHz while being probed with a 690 nm laser. The first and zero orders of diffraction are measured using a dual cell photodiode [113]. The relative displacements of the cantilever are reported to be measured with an accuracy on the order of 1 nm or less [113]. In addition to applications in AFM imaging, diffractive optomechanical sensors based on dynamic-mode cantilevers have been demonstrated as mass sensors [114]. The sensor presented by Ozturk et al. is based on the coupling of a diffraction grating with a magnetically actuated nickel cantilever [114]. The device is fabricated lithographically, and the cantilever is excited using a signal generator. The resonant behavior of the cantilever is monitored optically by a photodetector measuring the first order diffracted beam, any deflection of the cantilever as a result of mass loading causing a change in the diffracted light [114]. The device offers a resolution of 500 fg as well as not requiring vibration isolation, as is the case when using a laser Doppler vibrometer; furthermore, the signal-to-noise ratio between the devices is similar [114]. The disadvantages of these two sensors based on silicon cantilevers are the complex and expensive manufacturing methods and the complex read out of the sensors, which require complex electronics.

In addition to diffraction grating sensors, mechanical cantilever transducers have also been coupled with FBG-type sensors. Tian et al. [115] coupled an FBG sensor with a bimetal cantilever to create a temperature independent strain sensor. The cantilever is formed of two metals, one with a low coefficient of thermal expansion and one with a high coefficient of thermal expansion [115]. The FBG is attached to the side with the low coefficient of thermal expansion. The sensor is tested against an FBG that is not attached to a cantilever sensor. This sensor demonstrated a temperature sensitivity of 9.5 pm/°C while the cantilever-attached FBG showed a dependence on temperature of just −0.4 pm/°C [115]. Thus, through coupling the optical sensor—FBG with a mechanical transducer—bimaterial cantilever, cross-sensitivity with temperature was greatly reduced [115]. Cantilever/FBG sensors have also been demonstrated for civil engineering applications. Nazeer et al. have developed an FBG sensor for detecting the load induced on a cantilever plate [116]. Four FBG sensors are attached to the back of a 1 m^2^ cantilever plate. A load is applied to the plate at arbitrary locations across the beam [116]. Using an algorithm based on an interpolation of the strain information of the four FBG sensors, the position of the applied load can be determined within a 9% error in the 2D plane [116].

Abushagur et al. [117] have developed a sensor, based on a simple single beam cantilever, to distinguish between temperature and transversal force simultaneously [117]. A metal beam cantilever (metal ruler) was fixed at one end with a fiber containing the FBG, attached to the top side of the beam [117]. Forces applied to the free-end tip of the cantilever affect the FBG in a non-uniform way resulting in local wavelength shifts, whereas the entire spectrum of the FBG will respond to temperature, as a temperature gradient does not exist across the sensor [117]. This novel method discriminates between transversal force and temperature by analyzing the longer and shorter wavelengths (LW and SW, respectively) [117]; a sensitivity of 0.0712 nm/N for LW and 0.0573 nm/N for SW was demonstrated while the response for temperature was the same with a sensitivity of 0.0107 nm/°C [117]. The ability of an FBG sensor coupled with a cantilever transducer to act as a temperature insensitive detector was utilized by Guo et al. to form a liquid level sensor [118]. Their sensor is constructed from a high elastic steal bending cantilever beam with an FBG attached to the back side of the fixed end of the cantilever system [118]. The optomechanical cantilever FBG sensor operates in a similar manner to the other strain sensors already described; the bending of the cantilever with changing liquid level results in a broadening of the spectrum and a change in the reflected optical power [118]. For a 500 mm change in liquid level, there is only a 2% fluctuation in the measurement from 0 °C to 80 °C [118]. Another coupling of an FBG with a cantilever results in a device capable of the simultaneous detection of displacement and temperature [98]. With a similar configuration of an FBG attached to a cantilever, free at the opposite end, the device developed by Dong et al. can detect displacement with a sensitivity of 8.22 × 10^−4^ mm^−1^ and a temperature sensitivity of 8.86 × 10^−5^ (°C)^−1^ [98].

While cantilever sensors coupled with diffractive optical devices (diffraction gratings/FBGs) are prevalent in literature for classical optomechanical sensors, examples of a coupling of membrane-type sensors with optical devices has also been demonstrated. A number of different combinations with photonic crystal type optical sensors have been reported. Liu et al. present an optomechanical membrane resonating sensing system [119]. Their devices use a fiber-end facet and a graphene/Au membrane to act as partially reflective mirrors, forming an optomechanical cavity. The application of a current changes the resonance of the membrane, resulting in a variation in the optical output of the cavity [119]. Sensitivity of 7.91 and 18.04 Hz/mA^2^ has been reported monitoring first- and second- order vibrational modes, respectively [119]. Zhang et al. present an injection force sensor based on the combination of a membrane and a transmission phase grating [120]. The sensor combines two phase gratings, aligned vertically. When the injector is displaced, the upper grating position changes relative to the bottom grating, resulting in a change in the efficiency of the diffractive structure [120]. The system measures both the grating displacement and the displacement of the embryo membrane by assuming a spring constant of the structure and deriving the displacement based on the calculated change in the grating position [120]. Membrane transducers have also been coupled with photonic crystal sensors as demonstrated by Nazirizadeh et al. [121]. They present a PDMS membrane pressure sensor utilizing a photonic crystal slab. When the PDMS membrane is placed under pressure, it dilates, coming in contact with the inflexible photonic crystal slab [121]; this results in a circular shape output, the radius of which is a function of the pressure applied [121]. The device has demonstrated a sensitivity at 2 kPa of 1.25 mm/kPa and 0.17 mm/kPa at 8 kPa [121]. Photonic crystal sensors have also been coupled with cantilever sensors. Mai et al. present a static deflection type cantilever with a nanocavity and nano ring-type resonator [122]. When the cantilever deflects with loading, the resonant wavelength shift of the cavity is measured, and the sensitivity for the nano ring resonator formation is reported as being able to detect forces at 0.0757 μN [122]. Similarly, Tung et al. have coupled a 2D photonic crystal, positioned at the fixed end of a cantilever sensor [123]. When a load is applied to the free end, the lattice of the photonic crystal changes with a demonstrated sensitivity to strain of 0.95 pm/10^−6^ strain [123].

Optomechanical devices based on the combination of two or more classical mechanical or optical sensing principles invariably lead to an improvement in the overall quality of a sensing device when compared with the sensitivity of the mechanical or optical modes alone. In many cases, this improvement is a result of a direct improvement in device resolution [110,112], while in other it addresses issues of cross-sensitivity from other analytes or mechanical influences [114,118]. While the best-researched combination of a cantilever structure with a diffractive grating of FBG sensor has demonstrated significant potential for improvement in sensing technology for numerous analytes, early testing with photonic crystal and membrane-type structures, based mainly on detecting loading/force/strain analytes, shows great potential for functionalization as highly sensitive and dynamic devices.

## 5. Optical and Mechanical Sensors—Advantages, Challenges, and the Benefits of an Optomechanical Approach

Clearly, optomechanical sensors have been utilized as effective and novel transducers for many analytes and using many configurations. In this perspective, we propose that the optomechanical approach can offer significant benefits compared with the diffractive optical and mechanical approaches used as standalone methods. For this, it is important to analyze the advantages and the challenges that these approaches are bringing to sensing, but also to understand the benefit in their coupling together as optomechanical sensors.

### 5.1. Optical Sensors—Advantages and Challenges

The general advantages of optical sensors include the absence of electrical signals and, thus, they have the ability to work in a hazardous environment, ease of operation, low power consumption, and rapid real-time response. The versatility of optical phenomena allows choosing from a wide range of sensing methods and light-detection techniques, i.e., examination of the reflection or transmission spectrum or intensity measurement for a given wavelength. In some instances, it is also possible to observe analyte-induced changes with the naked eye (as the device color change), significantly simplifying the sensors’ operation.

The main challenge with optical sensors is their sensitivity to various environmental factors, such as temperature, humidity, and change in the refractive index of the surrounding medium caused by the presence of chemical analytes. Specific types of optical sensors possess additional capabilities but may also have certain limitations.

Thus, the holographic sensors (which are diffractive optics sensors) are typically compact in size and can be miniaturized [73]. Nevertheless, a relatively small sensing area, restricted by the spot size of the interfering beams used for fabrication, could be an obstacle in some cases. A solution to this challenge could be the use of arrays of surface relief gratings to enable easy copying of a structure once fabricated, which could benefit mass production. They can also be more easily functionalized as the grating is at the surface. However, one should have in mind that, sometimes, fabricating deep surface modulation—which will contribute to higher sensitivity—could be challenging. Another option for holographic sensors is the use of volume gratings. These gratings, in addition to changing the material’s effective refractive index, could also swell upon exposure to external factors. Moreover, the light is diffracted only in one diffraction order, enabling theoretically up to 100% of incident light to be redirected, thus significantly limiting power loss. Both features mentioned above significantly extend the detection sensitivity of volume gratings. In the case of sensors operating in reflection, observing the image displayed by the hologram with the naked eye and detection based on color changes is possible. The key challenges here are achieving high hologram brightness and the angular selectivity, which means that the angle of view could be narrow. As a result, the color change, perceived as a sensing response, could be caused by a variation in the viewing angle, not by the actual presence of the target analyte [25,124,125].

Optical fibers are frequently chosen for detection due to several advantages, including low weight, ability to operate at standard telecommunication wavelengths, and ease of multiplexing for sensor arrays fabrication, enabling measurement of multiple parameters and enhanced selectivity. In addition, vapor mists and dust in the surroundings cause no optical energy losses. However, fibers implementation in a working environment requires special care, as they are highly susceptible to bending and stretching. Moreover, the optical waveguide’s dispersion, birefringence, and nonlinear effects can affect the resulting output spectrum [126,127]. Interferometric fiber methods could offer high detection precision if extensive fringe analysis and phase retrieval processes are implemented. Nevertheless, the periodic output signal and demodulation of the interference spectrum remain the main challenges in these techniques. The Fabry–Pérot interferometer is highly sensitive to perturbations causing changes in its optical path length. In the case of the Mach–Zehnder interferometer, extraction of spectral peaks can be easily affected by errors due to multimode existence [85,128]. Fiber Bragg gratings (FBGs) have narrow spectral reflection bandwidth, improving resolution and achievable limit of detection. As the sensing response is encoded with wavelength, fluctuations in the intensity of the optical source do not interrupt proper detection. Notwithstanding, examination of the parameter signal encoded in the wavelength shift represents the key challenge here. The cross-sensitivity to temperature has a particularly strong influence on the measurement in FBG-based devices; therefore, thermal compensation is essential. Conventional FBGs cannot detect chemical analytes, and changes in refractive index in the surrounding medium is requiring additional cladding removal [129,130,131].

The photonic crystals have a relatively broad sensing area; thus, they have the potential to be easily integrated in lab-on-a-chip devices, and the ability to be used as colorimetric detectors. However, the angular dependence on the reflection spectrum requires maintaining a constant angle of incident light during measurements. Three-dimensional crystals, or so-called opal-like structures, are particularly attractive for gas detection due to their large porosity, thus enabling efficient permeability of chemical compounds into them. Unfortunately, they are also very susceptible to humidity. One-dimensional structures can be easily combined with optical waveguides, but special attention must be paid to possible strain. In 2D and 3D crystals, stimuli-responsive materials can be introduced as defects to improve detection performance further. However, it should also be noted that more structural dimensions usually indicate more complex and time-consuming fabrication methods [132,133,134].

Table 8 summarizes the primary advantages and challenges of diffractive optical sensors—holographic, fiber optic/FBG, and photonic crystals.

### 5.2. Mechanical Sensors—Advantages and Challenges

In terms of mechanical sensors that lend themselves to coupling with optical transducers, as seen thus far, cantilever sensors are prevalent. As discussed in Section 2, the cantilever sensors are, typically, used in one of the following two configurations: the static deflection or dynamic mode [44]. In both cases, the primary advantage is their simplicity in terms of mechanism of operation which is well understood from both theoretical and experimental principles. They also share common advantages in terms of the possibility of miniaturization, ease of mass production (if one considers the fact that for micro-cantilevers, as the AFM tips, the classical lithographic methods are typically used), and fast response time [44]. The disadvantages will be their functionalization, which is not always a straightforward process, or the incorporating of diffractive optics elements, as this will requiring micro-machining or precise and expensive setups (e.g., e-beam lithography).

Static deflection-mode cantilevers have the advantage of the possibility of operation without any actuation, electrical, magnetic, or otherwise [52]. They can also be used in micro (e.g., AFM tips) or macro (few cm long) configuration. As a result, in theory, their operation, much like the optical sensors discussed, is unaffected by electromagnetic fields. While the static cantilever can operate in isolation without additional hardware for activation, readout typically can require additional equipment, particularly for the microcantilever ones. Typically, this implies employing the optical level technique that requires a laser- and position-sensitive photodetector [135]. Alternatively, the cantilever can be fabricated from a piezoresistive material, requiring an electronic readout setup [136]. The additional hardware required increases cost of operation significantly.

Perhaps the biggest advantage of this method of sensing is its adaptability. The basic platform, based on a bilayer system, can be adapted for almost any analyte. The same can be said for dynamic-mode cantilever sensors. This adaptability, however, leads to possible complications. Firstly, in terms of fabrication, creating layers with desired sensing and mechanical properties conducive to high-quality sensors can require complex and potentially costly fabrication methods [44,52].

An issue that is common with almost every transducer is cross-sensitivity. Static deflection bilayer cantilevers, for example, will always suffer from some degree of cross-sensitivity with temperature and thermal drifts, unless the coefficients of thermal expansion of the bilayer are very similar. Therefore, in some cases additional coatings are used to address this [137]. Another way in which issues with selectivity are addressed for both static- and dynamic-mode cantilevers is through the use of an array system. Thus, each cantilever in the array can be differently functionalized to react to specific analytes [138]. While the use of an array of cantilevers can allow for the discerning of different analytes by one sensing system, it does come with additional challenges in terms of readout. This is particularly true for optical readout, which would require more complicated laser probing and photodetectors or one photodetector with multiple lasers switched on sequentially—this is a complex arrangement with difficulties in alignment and beam overlapping [139].

One distinct advantage of dynamic-mode cantilever sensors is that they have greater sensitivity to mass than static-mode sensors [140]. The disadvantage of dynamic-mode sensors is that they require some form of actuation—electrical, magnetic, electrostatic, acoustic, or piezo-acoustic [136]. However, as the cantilever readout is based on mass loading, the dynamic mode does not require a bilayer system (as the static mode does) with one layer functionalized for sensitivity to the target analyte [139]. This can potentially avoid complications in fabrication in terms of creating a thin, high-quality coating on a bulk substrate. In liquids, however, the resolution of dynamic-mode cantilevers is reduced due to the inherent damping of the liquid [44].

Despite the challenges listed, cantilever sensors have been demonstrated as an extremely useful base platform upon which more advanced sensing systems can be developed. While this perspective has demonstrated their utility in optomechanical sensing, cantilever sensors have seen extensive use in MEMS devices [141,142]. This is perhaps the most advantageous aspect of cantilever sensors—their ability to be integrated in numerous configurations and techniques of sensing systems.

The other mechanical transducer discussed in this perspective that has been identified as conducive with optical sensor coupling is the membrane sensor. Distinct from cantilever sensors, membrane transducers have a great number of sensing principles and configurations, making it difficult to list all the advantages and challenges associated with the method. This in itself is both an advantage and a challenge of the membrane sensing. While extremely adaptable for coupling with other technologies, pure mechanical membrane sensors, as such, do not exist, or at least have not garnered significant research interest. The coupling into another sensing system, commonly a capacitive sensor, is required for operation. In this perspective, membrane sensors in piezoresistive [62], capacitive [64], or potentiometric [65] configurations have been reported in the literature. All these devices presented highly sensitive qualities with relatively simple readout, but they require an electronic hardware of some kind to operate. Like the cantilever sensors, membrane devices have the advantage of being simple in their operation. One advantage they present over cantilever devices, specifically bilayer cantilever static sensors, is they do not require a bilayer or even necessarily a functionalized material. As a membrane is fixed at all ends, one can envisage, for example, a vapor sensor where the membrane inflates when the gas is present. While functionalization would not be required for operation, selectivity, as for the cantilevers arrays, could be achieved through an array of functionalized single layer membranes for different analytes. This system would, however, require coupling with an optical or electrical sensing system for readout.

The advantages and challenges of mechanical sensors are summarized in Table 9.

### 5.3. The Benefits of an Optomechanical Approach

Having examined the advantages and challenges of optical and mechanical sensors in isolation, we will now look at how the combination of both, in an optomechanical configuration, is a beneficial approach. Table 10 summarizes the advantages of coupling the technologies discussed in this perspective.

The coupling of optical and mechanical transducers not only enhances the sensitivity of both modes compared with the cases when they are used independently (i.e., in isolation). Additionally, this coupling can reduce cross-sensitivity and improve the operation mechanisms of the sensors, leading to easier readouts. Volume holographic gratings are very angularly selective [25]; as such, detuning from the Bragg angle results in a large variation in diffraction efficiency. In conventional holographic sensors, only the refractive index modulation or grating fringe spacing can change. When coupled with a cantilever sensor, the probe beam angle incident on the grating can effectively change [110,111]. This greatly enhances the sensitivity of the holographic sensor. Furthermore, the angular selectivity of the holographic grating allows for very small deflections of the cantilever sensor to be detected using relatively simple equipment (e.g., a laser and a power meter). For reflection gratings, fixing the hologram on a cantilever leads to a greater change in the fringe spacing of the grating than for a reflection grating sensor [111]. This leads to a large shift in the wavelength of the diffracted light.

The coupling of AFM cantilevers with diffraction gratings has improved sensor quality and resolution for both static- and dynamic-mode (AFM contact and tapping mode) cantilever sensors. [112,113]. For both dynamic and static cantilevers, along with an enhanced sensitivity, the coupling with a diffraction grating allows for a reduced sensitivity to vibrations. Laser pointing fluctuations allows for reduction in cross-talk between cantilever vibrations and the movement of the sensor tip [113].

As mentioned already, one of the primary issues facing all FBG sensors is the cross-sensitivity with the temperature. The coupling of an FBG with a cantilever greatly reduces this issue while maintaining sensitivity [115], and it has also led to a position-sensitive cantilever beam [116]. This novel configuration could potentially be of great interest in environmental-type sensors, with the potential to reduce cross-sensitivity and improve selectivity.

Photonic crystal sensors have been integrated into optomechanical devices for both cantilever and membrane-type sensors. These configurations are novel and require further investigation prior becoming commercially available. However, this coupling has significant potential as a highly sensitive strain sensor [121,122] with functionalization of the cantilever-style sensor highly possible [122]. Miniaturization is possible for both configurations [121,122].

A direct comparison between diffractive optomechanical sensors with optical and mechanical sensors is challenging as many different materials, configurations, and analytes have been investigated, making such a comparison difficult. Notwithstanding this challenge, Table 11 presents the performances of diffractive optomechanical, optical, and mechanical sensors for humidity and pressure along with their advantages and challenges.

A direct comparison between mechanical, optical, and optomechanical sensors is challenging (as is demonstrated by Table 11) due to the difficulty in finding sensors with similar materials, sensing mechanisms, and analytes. One advantage of optomechanical sensors is the coupling of two simply fabricated devices to form a more sensitive transducer. The simplicity of fabrication and readout is, obviously, a subjective matter. Much of the state of the art in sensing consists of devices with electronic components. This is the case for most membrane-type sensors, which operate as capacitive devices. Electronic-type sensors are intrinsically more complex in terms of fabrication than non-electronic devices and they will also require additional hardware for readout. In an optomechanical coupling, however, a membrane device can operate as a sensor without the requirement for inbuilt electronic components, as demonstrated by Nazirizadeh et al. [121]. In this device, while a quantitative measurement of pressure is possible using crossed polarization filters, the pressure can also be estimated by observing the device visually, i.e., a simple, low-cost readout method [121]. Similar can be said of the humidity sensor proposed by Grogan et al. [110]. Thus, while the humidity can be measured with high accuracy optically, a quick visual measurement is also possible. With a traditional holographic transmission grating, this is not possible. Optomechanical sensors can often operate with the same readout equipment as would be required for the optical sensor upon which the device is based. The holographic optomechanical cantilever sensor presented by Grogan et al. requires a laser and power meter for readout—as would be required by many holographic sensors—however, the Grogan et al. sensor offers a tenfold increase in sensitivity over cantilever or holographic sensors in isolation [110]. While direct comparison of sensitivities between different sensors is a challenge, a number of conclusions can be made. It can be seen that optomechanical coupling can possibly allow for more simply fabricated devices with simpler operation of otherwise more complex transducing methods, while maintaining device resolution and allowing for electromagnetic insensitivity.

The coupling of mechanical and optical sensors clearly has great benefits in improving sensitivity, reducing cross-sensitivity with other analytes, improving sensor operation, and creating novel configurations for potentially highly sought-after sensing parameters. However, there are challenges to this approach. The requirement of almost any optical sensor is for some type of photonic actuation/probing. This is also true for optomechanical sensors where a light source (laser, white light) is required for operation. Readout equipment will also be required, usually in the form of a photodetector or spectrometer. This is not a significant disadvantage as, for example, most state-of-the-art MEMS devices require more complex readout and operation equipment, while not having electromagnetic insensitivity which optomechanical sensors benefit from.

## 6. Conclusions

Optical and mechanical transducers have been prevalent in both commercial settings and research literature for over half a century. Such devices have been demonstrated as high resolution sensors across numerous fields. Since the late 1970s, electrical sensing devices have garnered significant interest, with MEMS sensors becoming commercially available in the late 1990s and early 2000s. Throughout this time, complex and compound configurations of electromechanical devices have been developed including MOEMS sensors as well as quantum effect-based sensing platforms. Despite the vast technological advancement presented by these technologies, research interest has persisted in simpler, classical diffractive optomechanical sensors. In this perspective, we have emphasized the efficacy of diffractive optomechanical sensing devices in providing high resolution, low-cost adaptable sensing platforms. These sensing platforms could be made through a combination of two or more well established technologies, e.g., one mechanical and one optical. The combination of these techniques enhances their sensitivity and exceeds the sensitivity of the individual optical and mechanical elements. While membrane-based optomechanical sensors have been presented, cantilever-based devices are prevalent among such configurations offering an adaptable platform upon which to build. The coupling of mechanical cantilever devices has been discussed for holographic diffraction gratings operating on both transmission and reflection, interdigital diffraction gratings for AFM type devices and for FBGs. Such cantilever-based sensors could operate in both dynamic and static deflection modes. Photonic crystal sensors could also be coupled with static deflection cantilevers, with a similar enhancement in device sensitivity being observed. While at present MEMS-type sensors dominate much of the current state-of-the-art and literature, the interest in optomechanical sensors has persisted as they offer a number of advantages as low cost, often simple fabrication, well understood and demonstrated theoretical operating principles, electromagnetic insensitivity, and adaptability as sensing platforms for multiple analytes. As a result, it is feasible to expect that optomechanical sensing devices with integrated diffractive optical element(s) will have a significant part in the next generation of state-of-the-art sensors with high resolution.

## Figures and Tables

**Table 1:** Examples of current state of the art in classical optomechanical sensing.

Sensor Type	Analyte	Sensitivity	Ref.
Micro-Optomechanical Cantilever	Thermal	0.035 mW^−1^ Resolution: 2 μK	[32]
Palladium-based Optomechanical Cantilever	Hydrogen	Detection Limit < 250 ppm	[33]
Nanophotonic Sensor based on Microcantilever	Chemical Sensor	Minimum detectable displacement—0.6 μm (water) 0.812 μm (air)	[34]
Optomechanical MEMS Interferometer	Acceleration	893.23 μm/g (mechanical) 15,874 V/g (voltage)	[35]
Optomechanical Bragg Reflector	Force	6.5 MHz/N	[36]
Bi-Material Micro-Cantilever	Temperature	28.4 μm/μW	[37]

**Table 2:** Examples of cantilever sensors operating in static and dynamic mode.

Sensor Type	Sensor Principle	Analyte	Sensitivity	Ref.
Sensing Bilayer on Microcantilever	Static (Piezoresistor)	Trace Explosives	Exhibited Response to 0.1 ppb TNT Vapour	[45]
Bimaterial Microcantilever	Static	Temperature Changes in Brown Adipocytes	Possible to measure < 1 K	[46]
Microcantilever Array	Static	Gas (Ethanol/Butane)	100–1000 ppm	[48]
Magnetically Actuated Resonant Cantilever	Dynamic	Gas	1.2 ppm (Limit of Detection for Toluene)	[49]
Silicon Resonant Cantilever	Dynamic	Airborne Nanoparticles	Mass Sensitivity—10 Hz/ng	[50]

**Table 3:** Examples of membrane-style sensors.

Sensor Type	Sensor Principle	Analyte	Sensitivity	Ref.
Membrane Encapsulated ZnO Nanowires	Membrane acts as selective barrier to MOF sensor	Gas	Tested for 10, 30 & 50 ppm H_2_	[61]
Cross Beam Membrane and Peninsula Diaphragm	Piezoresistive Sensor	Pressure	25.7 mV/kPa	[62]
Flexible Capacitive Sensor	Nanofiber Membrane with changing electrode distance	Pressure	~0.99/kPa	[63]
PVDF/Graphene Membrane	Capacitive Sensor based on changing dielectric properties	Humidity	Depending on Material: 0.0099–0.0463 pF/%RH	[64]
PVC Membrane Sensor	Potentiometric Sensor	Anti-Epileptic Drug Levetiracetam	Detection Limit: 6.31 × 10^−6^ molL^−1^	[65]

**Table 4:** Current state of the art of holographic diffraction sensors.

Holographic Material	Analyte	Principle	Readout	Sensitivity	Ref.
Acrylamide Photopolymer	Temperature	Volume change in material/Decrease in refractive index	Peak Wavelength Shift	−0.743 to −2.323 nm/°C (depending on relative humidity)	[76]
Acrylamide/N-Isopropanol acrylamide Photopolymer	Temperature	Change in grating thickness/Refractive index modulation	Diffraction Efficiency (DE)/Spectral Change	24% decrease (NIPA) 2% decrease (AA) in DE at 60 °C	[77]
Sylgard 184 PDMS	Hydrocarbons (Gas)	Change in fringe spacing in presence of analyte	Reflection hologram colour change	Detection limit of ~5% (*v*/*v*)	[78]
Acrylamide Photopolymer	Humidity	Material volume change	Shift in peak wavelength	114 ± 3 nm/mg	[79]
PHEMA Hydrogel	pH	Swelling and shrinking resulting in change of grating spacing	Shift in peak wavelength	From pH 4–pH 7, max. shift at steady state >150 nm in linear region	[80]
Acrylamide Photopolymer	Pressure	Material volume change	Shift in peak wavelength	4.9 × 10^3^ Pa/nm	[81]

**Table 5:** Current state of the art of Fiber Optic Sensors.

Sensor Type	Analyte	Sensitivity	Ref.
Michelson Interferometer	Ethanol & Benzene	Tested for Ethanol: 1611–32,210 ppm Benzene: 964–19,290 ppm	[86]
Fabry–Perot Interferometer	High Temperature Sensing	13.6 pm/°C	[87]
Mach–Zehnder Interferometer	Methane	Transmission spectrum increases 1.033 dB from 0–34.3% Methane	[88]
Absorption (Refractive index change)	Acetone	14.3% greater than ammonia & 7.4% greater than ethanol	[89]
Nanopatterned Fiber-Tip	Ethylene	Detection Limit ~4.7 ppm	[90]
Fabry–Perot Interferometer	Pressure and Temperature	Pressure: −36.93 nm/MPa Temperature: 10.29 nm/°C	[91]

**Table 6:** Sample of the Current State of the Art of Photonic Crystal Sensors.

Sensor Type	Analyte	Sensitivity	Ref.
2D Slotted Photonic Crystal Cavity-Based Sensor	Methane	614 nm/RIU	[104]
Photonic Crystal Hydrogel Matrix	Ionic Strength (pH)	0.03 logarithmic units (From 10^−4^–10^−2^ mol∙L^−1^)	[105]
Doped PDMS Photonic Crystal Sensor	Solvents	2 nm shift for 1% (*v*/*v*) CH_3_OH	[106]
Photonic Crystal Cavity	Hydrogen Sulfide	2.3 × 10^5^ nm/RIU	[107]
Photonic Crystal Fiber-Based Sensor	Cancer Cells	Detection Limit of 0.024	[108]

**Table 7:** Examples of Classical Diffractive Optomechanical Sensors.

Sensor Type	Mechanical Principle	Optical Principle	Analyte	Sensitivity	Ref.
Bimaterial Cantilever	Static Deflection Cantilever (fixed end deflection)	Holographic Transmission Diffraction Grating	Relative Humidity (RH)	Mechanical: 1% RH Optical: 0.1% RH	[110]
Bimaterial Cantilever	Static Deflection Cantilever (free cantilever bending)	Holographic Reflection Diffraction Grating	Not Functionalized	Average: 22.42 nm/degree	[111]
AFM Cantilever	Static Deflection in contact with sample (AFM Contact mode)	Interdigital Optical Diffraction Grating	AFM Measurements	More sensitive than optical lever AFM configuration	[112]
AFM Cantilever	Dynamic-Mode Cantilever (AFM Tapping mode)	Interdigital Diffraction Grating	AFM Measurements	Can detect Displacements of 1 nm or less	[113]
Nickel Cantilever	Dynamic-Mode Cantilever	Diffraction Grating	Mass	Resolution: 500 fg	[114]
Fiber Bragg/Bimaterial Cantilever	Static Deflection Cantilever	Fiber Bragg Grating	Strain	N/A (Paper focuses on temperature independence of sensing platform)	[115]
Fiber Bragg Grating	Large Cantilever Plate	Fiber Bragg Grating	Cantilever plate load	Load position estimated within 9% accuracy	[116]
Cantilever Beam with Single Fiber Bragg Grating	Static Deflection Cantilever	Fiber Bragg Grating	Temperature and Transversal Force	Responsivity Ratio: 0.0107 nm/°C	[117]
Fiber Bragg Grating based on a Bending Cantilever Beam	Bending Cantilever Beam	Fiber Bragg Grating	Liquid Level	For liquid level variation of 500 mm (from 0–80 °C) < 2% fluctuation in measured level	[118]
Cantilever-Based Fiber Bragg Sensor	Static Deflection Cantilever	Fiber Bragg Grating	Displacement and Temperature	Displacement: 8.22 × 10^−4^ mm^−1^ Temperature: 8.86 × 10^−5^ (°C)^−1^	[98]
Nano–Optomechaical Resonator Based Membrane	Membrane	Fabry—Perot Cavity	Current	7.91 & 18.04 Hz/mA^2^ monitoring first and second order vibrational modes	[119]
Micrograting-Based Injection Force Sensor	Membrane	Transmission Phase Grating	Force	Not Reported	[120]
Photonic Crystal Slab	Membrane	Photonic Crystal	Pressure	For 2 kPa: 1.25 mm/kPa For 8 kPa: 0.17 mm/kPa	[121]
Photonic Crystal Resonators for Silicon Microcantilevers	Static Deflection Cantilever	Photonic Crystal	Force and Strain	Minimum Detectable: Force: 0.0757 μN Strain: 0.0023%	[122]
Single—Defect Photonic Crystal Nanocavity	Static Deflection Cantilever	2D Photonic Crystal	Strain	Measured Shift: 0.95 pm/10^−6^ strain	[123]

**Table 8:** Summary of main Advantages and Challenges of Diffractive Optical Sensors.

Sensor Type	Advantages	Challenges
Holographic	Electromagnetic insensitivity, compact size, simple fabrication, possibility for mass production, use as colorimetric devices	High angular dependence, cross-sensitivity with temperature and humidity, small sensing area
Fiber Optic/FBG	Electromagnetic insensitivity, lightweight, operate at telecommunications wavelengths, light does not propagate in free space	Easily deformable affecting output, cross-sensitivity to temperature, demodulation of interference spectrum, requires additional cladding
Photonic Crystals	Electromagnetic insensitivity, broad sensing area, ease of interrogation, 3D structures’ porosity advantageous for gas sensing, ability for use as colorimetric devices	Angular dependence requires maintaining incident angle throughout measurement, cross-sensitivity with humidity (3D in particular), cross-sensitivity with strain and temperature

**Table 9:** Summary of main Advantages and Challenges of Mechanical Sensors.

Sensor Type	Advantages	Challenges
Static Deflection Cantilever	Mass production and miniaturization possible, can operate without actuation, electromagnetic insensitivity, highly adaptable platform, well-defined theoretical models for parameters affecting sensitivity.	Cross-sensitivity with temperature, could requires hardware (see optical lever method) for readout, complex array format required for selectivity.
Dynamic Resonance Cantilever	Mass production and miniaturization possible, very sensitive to mass, no direct requirement for functionalizing bilayer, highly adaptable platform, well-defined theoretical models for parameters affecting sensitivity.	Lower Q factor when operating in liquid, requires actuation of some kind for operation, often affected by electromagnetic and temperature cross-sensitivity, complex array format required for selectivity.
Mechanical Membrane	Adaptable sensing platform, functionalization not necessarily required, generally simple principle of operation/fabrication.	Cannot operate in isolation, i.e., coupling with another sensing method generally required, more research required on optimized parameters and mechanical properties for highest sensitivity.

**Table 10:** Benefits of an Optomechanical Approach.

Mechanical Transducer	Optical Transducer	Benefits of Coupling
Static Deflection Cantilever	Holographic Diffraction Grating	Improves resolution of detection of cantilever deflection. In transmission mode allows for Bragg angle detuning—not possible in conventional holographic sensor. In reflection mode, cantilever bending allows for bigger change in grating fringe spacing than conventional reflection sensing.
	Interdigital Finger Diffraction Grating	Enhances sensitivity compared with optical lever techniques for AFM cantilever sensing. In addition, insensitive to pointing fluctuations in laser and thermally induced mechanical vibrations. Simpler alignment when used in an array.
	Fiber Bragg Grating	Reduced cross-sensitivity with temperature. Can be coupled with cantilevers in novel configuration to detect load position on cantilever. Ability to discern between transversal force and temperature.
	Photonic Crystal	Photonic crystal capable of detecting loading on cantilever with linear response. Can be integrated on small structures. Smaller effect of noises on the sensor.
Dynamic Resonance Cantilever	Interdigital Finger Diffraction Grating	Lower signal-to-noise ratio, reduced cross-talk between cantilever vibrations and tip movement.
	Diffraction Grating	Immunity to environmental noise. Measurements can be taken with a single photodetector due to beams with slightly different resonance frequency.
Mechanical Membrane	Fabry—Perot Cavity	Enhanced sensitivity. Low power consumption. Compact size. Adaptable sensing platform, possible functionalization for multiple analytes.
	Transmission Grating	Allows for coupled measurement of both grating and membrane deformation position.
	Photonic Crystal	Novel configuration, further investigation required. Non-contact sensor. Miniaturization Possible

**Table 11:** Comparison of Diffractive Optomechanical, Mechanical, and Optical Sensors.

Sensor Type	Analyte	Sensitivity	Advantages	Challenges	Ref.
Mechanical (Cantilever)	Humidity	3.7 MHz/%RH	Low cost, simple fabrication	Fabrication reproducibility quite low	[143]
Mechanical (Membrane)	Humidity	Depending on Material: 0.0099–0.0463 pF/%RH	High sensitivity, linear response	Complex fabrication and readout requirements	[64]
Optical (Holographic)	Humidity	114 ± 3 nm/mg	Low cost, size, simple readout	Cross-sensitivity with temperature	[79]
Optomechanical (Cantilever + Holographic Diffraction Grating)	Humidity	LOD: 0.1% RH	Low cost, simple readout, user friendly	Cross-sensitivity with other gaseous analytes	[110]
Mechanical (Membrane)	Pressure	~0.99/kPa	Fast response, loading/unloading stability	Complex fabrication and readout	[63]
Optical (Fiber Optic)	Pressure	−36.93 nm/MPa	Simultaneous temperature measurement, compact structure	Complex fabrication and readout	[91]
Optical (Photonic Crystal)	Pressure	26.1 nm/Gpa	Good quality factors, perfect linear relationship between cutoff wavelength and pressure	Proposed Sensor	[144]
Optomechanical (Membrane + Photonic Crystal)	Pressure	For 2 kPa: 1.25 mm/kPa For 8 kPa: 0.17 mm/kPa	Transparent, miniaturization possible	Complex Readout	[121]

## Data Availability

No new data were created.
